# The prognostic significance of PFS24 in follicular lymphoma following firstline immunotherapy: A combined analysis of 3 CALGB trials

**DOI:** 10.1002/cam4.1918

**Published:** 2018-12-21

**Authors:** Frederick Lansigan, Ian Barak, Brandelyn Pitcher, Sin‐Ho Jung, Bruce D. Cheson, Myron Czuczman, Peter Martin, Eric Hsi, Heiko Schöder, Scott Smith, Nancy L. Bartlett, John P. Leonard, Kristie A. Blum

**Affiliations:** ^1^ Dartmouth College Norris Cotton Cancer Center Lebanon New Hampshire; ^2^ Alliance Statistics and Data Center Duke University Durham North Carolina; ^3^ MedStar Georgetown University Hospital Washington District of Columbia; ^4^ Celgene, Inc. Morristown New Jersey; ^5^ Weill Medical College of Cornell University New York New York; ^6^ Cleveland Clinic Foundation Cleveland Ohio; ^7^ Memorial Sloan Kettering Cancer Center New York New York; ^8^ Alliance Protocol Office University of Chicago Chicago Illinois; ^9^ Washington University School of Medicine St. Louis Missouri; ^10^ Emory University School of Medicine Atlanta Georgia

**Keywords:** biologic agents, early progression, follicular lymphoma, immunotherapy, PFS24

## Abstract

Follicular lymphoma (FL) patients treated with firstline R‐CHOP who experience progression of disease (POD) within 2 years have a shorter survival than those who do not have POD within 2 years. Whether this observation holds for patients treated initially with biologic immunotherapy alone is unknown. We performed a retrospective analysis of 174 patients pooled from three frontline rituximab (R)‐based nonchemotherapy doublet trials: R‐galiximab (Anti‐CD80, CALGB 50402), R‐epratuzumab (Anti‐CD22, CALGB 50701), and R‐lenalidomide (CALGB 50803) to determine outcomes of early progressors and risk factors for early POD, defined as progression within 24 months from study entry. Twenty‐eight percent (48/174) of patients had early POD. After adjusting for the Follicular Lymphoma International Prognostic Index (FLIPI), patients with early POD from study entry had a worse OS compared with patients who did not progress within 2 years (HR = 4.33 (95% CI 1.50‐12.5), *P* = 0.007). For early POD, the 2‐year survival was 80% vs 99% for nonearly POD, and the 5‐year survival was 74% vs 90%, respectively. These findings suggest that the adverse survival of patients with early POD may be independent of initial treatment modality.

**Key Points**

In this combined analysis of three CALGB clinical trials, patients with early progression of follicular lymphoma following frontline immunotherapy doublets are at increased risk of death. PFS24 is a prognostic marker for overall survival in patients treated with rituximab‐containing immunotherapy.

## 1 INTRODUCTION

The prognosis for patients with follicular lymphoma (FL) has improved significantly since the introduction of rituximab, but there is still a subset of patients with a worse prognosis. The follicular lymphoma international prognostic index (FLIPI),^1^ FLIPI‐2,^2^ and the molecular FLIPI (m7‐FLIPI)^3^ are clinical and biologic prognostic indices that have been associated with 5‐ and 10‐year OS in newly diagnosed FL patients and have been traditionally used to estimate survival in newly diagnosed patients. Recently, several studies have also identified early relapse after firstline chemotherapy to be associated with poor OS.

In the National LymphoCare Project, progression of disease (POD) within 24 months of diagnosis was recently identified as an important prognostic factor for patients with FL treated with firstline R‐CHOP (cyclophosphamide, doxorubicin, vincristine, and prednisone).^4^ Progression of disease (POD) within 2 years of diagnosis was associated with and increased risk of death [hazard ratio (HR) 6.4] and a 50% 5‐year overall survival (OS), compared to a 90% 5‐year OS in patients who did not relapse within 2 years. This finding was validated in patients treated with rituximab, fludarabine, mitoxantrone, and dexamethasone (R‐FND).^4^ The German Low‐Grade Lymphoma study group and the British Columbia Cancer Agency also validated this finding among R‐CHOP and R‐CVP‐treated patients who progressed within 2 years of diagnosis.^5^ Taken together, these three studies confirm that progression within 2 years from diagnosis is associated with inferior OS in FL patients treated with rituximab‐containing combination chemotherapy. As a result of the similar findings from these trials, a large retrospective international study validated event‐free survival (EFS) at 24 months from diagnosis as relevant surrogate endpoint for OS in patients with FL initially treated with R‐containing chemotherapy.^6^

Not all patients require or can tolerate chemoimmunotherapy, and there are acute and long‐term toxicities associated with traditional chemotherapy such as CHOP, FND, and bendamustine. Thus, studies have evaluated noncytotoxic strategies for initial treatment of patients with FL. For example, E4402 (RESORT) enrolled untreated low‐tumor burden FL to single‐agent rituximab for four doses followed by either re‐treatment rituximab (RR) as needed or maintenance rituximab (MR).^7^ This study demonstrated that rituximab administered in an RR strategy is as efficacious as MR in low‐tumor burden FL with a 3‐year PFS of 65% vs 73%, respectively.^7^ SAKK35/98 comparing short course and extended rituximab dosing reported a 66% 2‐year PFS in the extended dosing arm.^8^ In a study from the United Kingdom, FL patients were randomly assigned to watch‐and‐wait or rituximab induction plus MR for 2 years. The primary endpoint was time to next treatment, and rituximab‐treated patients were far less likely to require new therapy at 3 years (88% vs 46%).^9^ Whether the early event status such as the progression‐free survival at 24 months (PFS24) from these trials can inform subsequent overall survival in patients treated without chemotherapy is unknown.

The Cancer and Leukemia Group B (CALGB) (now known as Alliance for Clinical Trials in Oncology) conducted a series of phase II studies to explore novel frontline rituximab (R)‐based nonchemotherapy doublets for patients with previously untreated FL from 2005 to 2011.^10^, ^11^, ^12^ In CALGB 50402, 61 patients bulky stage II or stage III‐IV grade 1‐3a FL were treated with 4 weekly doses of rituximab and galiximab (anti‐CD80 monoclonal antibody) followed by four additional doses of the combination every 2 months. With this combination, the overall response (OR) rate was 72.1%, with 47.6% complete responses (CR) and median PFS of 2.9 years.^10^ In CALGB 50701, 59 patients with bulky stage II or stage III‐IV grade 1‐3a FL were treated with combined rituximab and epratuzumab (anti‐CD22 monoclonal antibody) utilizing a similar schedule to CALGB 50402 with 4 weekly doses of the combination followed by four additional doses every 2 months. With rituximab‐epratuzumab, the OR was 88.2% with 42.4% CRs and a median PFS of 3.5 years.^11^ Lastly, CALGB 50803 examined the doublet combination of rituximab and lenalidomide; however, unlike the previous two trials, this study restricted eligibility to those patients with previously untreated grade 1‐3a FL with FLIPI scores of 0‐2. In this study, rituximab was administered weekly for four weeks during cycle 1 and then every 2 months for 4 additional doses with lenalidomide 20 mg days 1‐21 of a 28‐day cycle for up to 12 cycles. In 65 patients treated with rituximab and lenalidomide, the ORR was 93%, CR was 72%, and median PFS is not reached.^12^ With these differing immunotherapy combination regimens in a similar previously untreated FL patient population, the 2‐year PFS were 58%, 74%, and 86%, for CALGB 50402, 50701, and 50803, respectively.

In the current analysis, we performed a retrospective pooled analysis of patients enrolled on these 3 phase II trials, and evaluated if PFS24 could be used as a prognostic tool after initial biologic treatment. In addition, we identified risk factors for early progression of disease after treatment with these biologic doublets.

## 2 METHODS

Early POD was defined as progression within 24 months from study entry. Patients with early POD or non‐progressors with at least a two‐year follow‐up from study entry were included. Clinical trials CALGB 50402, 50701 and 50803 had similar eligibility criteria: previously untreated follicular lymphoma, grade 1, 2, or 3a with stage III, IV, or bulky (single mass >7 cm) stage II disease, and ECOG PS 0 to 2. Of note, while CALGB 50402 and 50701 allowed patients with FLIPI scores of 0‐5, CALGB 50803 was restricted to patients with FLIPI scores of 0‐2.

Univariable and multivariable logistic regression modeling using forward selection was performed to identify predictors of early POD. Kaplan‐Meier (KM) method was used to estimate 2‐year and 5‐year overall survival probability.^13^ As POD status is not known at time of study entry or diagnosis, to avoid a 2‐year survival bias for the reference group, survival is measured from a risk‐defining event: date of POD for early progressors and two years following study entry or diagnosis for the reference group. Hazard ratios (HR) and 95% CI were calculated using a univariable and multivariable Cox regression model adjusting for FLIPI. Because the definition of early POD in our analysis was time of *study entry* onto these therapeutic trials until disease progression, and was not the same definition as early POD used in the National LymphoCare, R‐FND and German Low‐Grade Lymphoma trials, which was time from *diagnosis* until progression, we also determined the association of POD within 24 months from the *diagnosis* of follicular lymphoma with survival in patients on these 3 CALGB trials.

We examined risk factors for age >60 years, hemoglobin <10 g/dL, number of lymph node sites >4, stage III/IV, lactate dehydrogenase (LDH) above normal, bone marrow involvement, lymph node size >6 cm, beta‐2 microglobulin >normal, and grade 3a disease. In addition, male or female sex, B symptoms, and albumin <3.5 were analyzed. Continuous variables white blood cell count (WBC) at diagnosis, absolute lymphocyte count (ALC), and absolute monocyte count (AMC), and ALC/AMC ratio were also considered. Lastly, we examined pathology features Ki‐67, CD68, FOXP3, PD1, PDL1, and interfollicular CD10 as done by Sohani et al.^14^

Using these risk factors, we developed a multivariable logistic model for early progression. Due to the limited sample size, a cross‐validation method (fivefold) was used for validation. Once this cross‐validation procedure was complete, the final logistic model was fitted using the whole data set.

Data collection and statistical analyses were conducted by the Alliance Statistics and Data Center. All analyses were based on the study database frozen as follows: CALGB 50402 on April 4, 2011, 50701 on April 17, 2012, and 50803 on May 29, 2014.

## 3 RESULTS

### 3.1 Patient characteristics

Sixty patients on CALGB 50402, 57 from CALGB 50701, and 57 from CALGB 50803 were included. Patient characteristics from the individual studies are shown in Table 1. The median age was 54, and 49.4% were male. For the entire group, FLIPI low, intermediate, and high was 24%, 52% and 24%, respectively. Of note, CALGB 50803 had only 3.5% high‐risk FLIPI since the study was designed to exclude high‐risk FLIPI patients. Median follow‐up time was 5.4 years (range 0‐10.1 years) and was shortest in the CALGB 50803 study, 4.5 years (range 0.9‐5.5 years). The median time from diagnosis to study entry was 1.94 months (0.20‐115 months).

**Table 1:** Patient characteristics

Variable	Overall N = 174	50402 N = 60	50701 N = 57	50803 N = 57	*P*‐value*
Sex					
Male	86 (49.4%)	36 (60.0%)	23 (40.4%)	27 (47.4%)	0.10
Female	88 (50.6%)	24 (40.0%)	34 (59.7%)	30 (52.6%)	
Age (years)					
Median (Range)	54 (22‐90)	57 (22‐85)	54 (32‐90)	52 (32‐79)	0.25
FLIPI					
Low	42 (24.4%)	12 (20.7%)	13 (22.8%)	17 (29.8%)	<0.01†
Intermediate	89 (51.7%)	25 (43.1%)	26 (45.6%)	38 (66.7%)	
High	41 (23.8%)	21 (36.2%)	18 (31.6%)	2 (3.51%)	
Early progression					
No	126 (72.4%)	35 (58.3%)	42 (73.7%)	49 (86.0%)	0.0036
Yes	48 (27.6%)	25 (41.7%)	15 (26.3%)	8 (14.0%)	
Median follow‐up in years (Range)	n = 185	n = 61	n = 59	n = 65	<0.0001†
	5.4 (0.0‐10.1)	6.7 (0.0‐10.1)	6.3 (0.3‐8.1)	4.5 (0.1‐5.5)	
Median time diagnosis to enrollment in months (Range)	n = 169 1.94 (0.20‐115)				

* Compares variables across studies

† Statistically significant, α = 0.05

Of the 174 patients, 48 (28%) had a relapse of lymphoma within 24 months of study entry. In our secondary analysis using time from diagnosis, 29 of 171 (17%) patients had relapse of lymphoma within 24 months of diagnosis. Three patients did not have the diagnosis date available.

Of the 48 early progressors, the median age was 57, 40.4% were intermediate‐risk FLIPI, 46.8% were high‐risk FLIPI, and 38.3% had bulky disease ≥7 cm. Twenty‐five (41.7%), 15 (26.3%), and 8 (14%) of the early progressors came from CALGB 50402, 50701, and 50803 studies, respectively. The median follow‐up time of the early progressors was 5.1 years (range 0‐8.1 years) compared to 5.4 years (range, 0‐10.1 years) for the entire cohort, which is likely a reflection of overall survival. Additional patient characteristics of early progressors are shown in Table 2.

**Table 2:** Characteristics of patients with early progression and univariable analysis

Variable	Overall	Early progression		OR (95% CI)	*P*
		No	Yes		
Age (years)	n = 174	n = 126	n = 48		
Median	54	53	57	1.03 (1.00, 1.06)	0.0300
Range	(22‐90)	(22‐83)	(32‐90)		
Age (years)					
0‐59	121 (69.5%)	94 (74.6%)	27 (56.3%)	‐	0.0187
60+	53 (30.5%)	32 (25.4%)	21 (43.8%)	2.28 (1.14, 4.59)	
Sex					
Male	86 (49.4%)	53 (42.1%)	33 (68.8%)	3.03 (1.50, 6.13)	0.0017
Female	88 (50.6%)	73 (58.0%)	15 (31.3%)	‐	
Hemoglobin (g/dL)					
<10	5 (2.87%)	1 (0.79%)	4 (8.33%)	11.36 (1.24, 104)	0.0209
≥10	169 (97.1%)	125 (99.2%)	44 (91.7%)	‐	
Number of nodal sites					
≤4	70 (40.5%)	58 (46.4%)	12 (25.0%)		0.0102
>4	103 (59.5%)	67 (53.6%)	36 (75.0%)	2.60 (1.24, 5.45)	
Stage of Disease					
Stage I‐II	11 (6.36%)	8 (6.35%)	3 (6.38%)	1.01 (0.26, 3.96)	1.0000
Stage III‐IV	162 (93.6%)	118 (93.7%)	44 (93.6%)	‐	
LDH					
Below ULN	154 (88.5%)	117 (92.9%)	37 (77.1%)	‐	0.0035
Above ULN	20 (11.5%)	9 (7.14%)	11 (23.0%)	3.86 (1.49, 10.0)	
Bone Marrow involvement					
No	86 (49.4%)	64 (50.8%)	22 (45.8%)	‐	0.5586
Yes	88 (50.6%)	62 (49.2%)	26 (54.2%)	1.22 (0.63, 2.38)	
Node Size (cm)					
<7	130 (77.8%)	101 (84.2%)	29 (61.7%)	‐	0.0017
≥7	37 (22.2%)	19 (15.8%)	18 (38.3%)	3.30 (1.53, 7.09)	
Beta‐2‐Microglobulin					
Below ULN	30 (60.0%)	27 (62.8%)	3 (42.9%)	‐	0.4161
Above ULN	20 (40.0%)	16 (37.2%)	4 (57.1%)	2.25 (0.45, 11.4)	
Grade 3a Disease					
No	160 (94.1%)	114 (92.7%)	46 (97.9%)	3.63 (0.45, 29.5)	0.2874
Yes	10 (5.88%)	9 (7.32%)	1 (2.13%)	‐	
B symptoms Present					
No	153 (91.6%)	111 (91.0%)	42 (93.3%)	1.39 (0.37, 5.22)	0.7611
Yes	14 (8.38%)	11 (9.02%)	3 (6.67%)	‐	
Albumin (g/dL)					
<3.5	10 (8.70%)	3 (3.95%)	7 (18.0%)	5.32 (1.29, 21.9)	0.0299
≥3.5	105 (91.3%)	73 (96.1%)	32 (82.1%)	‐	
WBC at baseline (×10^9^ per liter)	n = 171	n = 123	n = 48		
Median	6.3	6.4	6	1.09 (0.99, 1.21)	0.095
Range	(2.7‐77.8)	(2.7‐16.3)	(3.8‐77.8)		
ALC at baseline (×10^9^ per liter)	n = 168	n = 121	n = 47		
Median	1.38	1.4	1.26	1.16 (0.96, 1.39)	0.1169
Range	(0.43‐41.2)	(0.43‐10.1)	(0.45‐41.2)		
AMC at baseline (×10^9^ per liter)	n = 168	n = 121	n = 47		0.0595
Median	0.45	0.44	0.52	4.3 (0.94, 19.6)	
Range	(0.01‐3.04)	(0.01‐1.00)	(0.18‐3.04)		
ALC/AMC ratio	n = 168	n = 121	n = 47		
Median	3.07	3.15	2.78	1.03 (0.98, 1.08)	0.2963
Range	(0.83‐57)	(0.83‐57)	(0.85‐53)		
FoxP3					
Diffuse	94 (74.0%)	70 (76.9%)	24 (66.7%)	‐	0.3033
Follicular	3 (2.36%)	1 (1.10%)	2 (5.56%)	5.83 (0.51, 67.2)	
M	20 (15.8%)	14 (15.4%)	6 (16.7%)	1.25 (0.43, 3.62)	
Partial	10 (7.87%)	6 (6.59%)	4 (11.1%)	1.94 (0.51, 7.48)	
PD1					
≤5%	29 (31.2%)	22 (32.4%)	7 (28.0%)	3.82 (0.42, 34.8)	0.1685
6%‐33%	51 (54.8%)	34 (50.0%)	17 (68.0%)	6.00 (0.72, 50.1)	
>33%	13 (14.0%)	12 (17.7%)	1 (4.00%)	‐	
FLIPI					
Low	42 (24.4%)	36 (28.8%)	6 (12.8%)	‐	<0.0001
Intermediate	89 (51.7%)	70 (56.0%)	19 (40.4%)	1.63 (0.60, 4.44)	
High	41 (23.8%)	19 (15.2%)	22 (46.8%)	6.95 (2.41, 20.1)	
Ki67	n = 102	n = 72	n = 30		
Median	9.24	9.69	8.92	0.99 (0.95, 1.03)	0.5553
Range	(0.51‐67.5)	(0.51‐67.5)	(0.59‐39.9)		
CD10 (interfollicular)					
Negative	35 (35.4%)	30 (42.9%)	5 (17.2%)	‐	
Positive	64 (64.7%)	40 (57.1%)	24 (82.8%)	3.60 (1.23, 10.5)	0.0153
Diagnosis to Enroll (Month)	n = 169	n = 123	n = 46		
Median	1.94	2	1.87	1.00 (0.99, 1.03)	0.4231
Range	(0.20‐115)	(0.26‐115)	(0.20‐103)		
Survival Follow‐up (Years)	n = 174	n = 126	n = 48		
Median	5.4	5.5	5.1	0.77 (0.63, 0.94)	0.0093
Range	(0.0‐10.1)	(2.1‐10.1)	(0.0‐8.1)		

### 3.2 Early POD and survival

**Figure 1:**
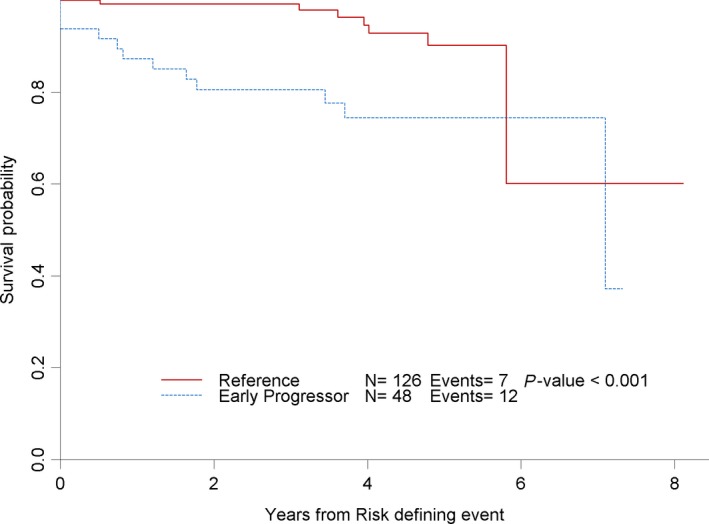
Overall survival (separate TIFF file)

Patients with early POD from study entry (n = 48) had a 2‐year OS of 80% (95% CI 66%‐89%) and 5‐year OS of 74% (95% CI 58%‐85%) compared to 99% and 90%, respectively, for patients without early POD (n = 126). Of the 48 patients with early POD, 12 (25%) have died during subsequent follow‐up, compared to 7 (5.6%) of the reference group. The Kaplan‐Meir curve of OS showed an inferior overall survival for patients with early POD compared to those not relapsing within 24 months(*P* < 0.001) [Figure 1]. Early POD from study entry was associated with an increased risk of death with a HR of 4.05 (95% CI 1.57‐10.5, *P* = 0.004). Even after adjusting for FLIPI, early POD from study entry was associated with an increased risk of death with an HR of 4.33 (CI: 1.50‐12.5, *P* = 0.007) [Table 3].

**Table 3:** Hazard ratio of overall survival

Overall Survival (Univariable)			Overall Survival (Multivariable)		
			FLIPI +Early progression		
	HR (95% CI)	*P*‐value		HR (95% CI)	*P*‐value
Early progression			Early progression		
No	‐	0.0039	No	‐	0.0068
Yes	4.05 (1.57, 10.5)		Yes	4.33 (1.50, 12.5)	
			FLIPI		0.6965
			Low	‐	
			Intermediate	0.79 (0.20, 3.20)	
			High	1.27 (0.31, 5.19)	

When early progression was defined from the time of diagnosis until progression, with 29 early progressors in this group compared to 142 patients without early progression, the results were similar. The 2‐ and 5‐year OS were 78% (95% CI: 57%‐89%) and 69% (95% CI 48%‐83%) for early POD from diagnosis compared to 2‐ and 5‐year OS of 99% and 93% in those patients without early progression from diagnosis. The HR for OS was 4.10 (95% CI 1.52‐11.0), *P* = 0.005 [Data not shown].

### 3.3 Risk Factors associated with early POD

For patients experiencing early POD, univariable analysis showed that clinical factors at study entry which predicted early POD and inferior overall survival were age, male sex, hemoglobin <10 g/dL, number of nodal sites >4, elevated LDH, FLIPI, lymph node size ≥7, albumin <3.5 g/dL, and interfollicular expression of CD10 by immunochemistry. By univariable analysis, hemoglobin, FLIPI 3‐5, and albumin were most highly associated with early progression, odds ratio of 11.36, 6.95, and 5.32, respectively (Table 2). In multivariable analysis, clinical factors predictive of early POD were male sex, albumin <3.5 g/dL, low absolute monocyte count (AMC), interfollicular CD10 expression, and FLIPI (Table 4). The biologic features of Ki‐67, CD68, FOXP3, PD1, and PDL1 expression did not correlate with early POD.

**Table 4:** Multivariable Analysis of risk factors associated with early POD

Variable	Description	Odds Ratio (95% CI)	*P*‐value
Sex	Male vs Female	7.93 (1.5‐41.66)	0.015
Albumin	<3.5 vs ≥3.5	17.51 (0.97, 315.27)	0.052
AMC	1 unit decrease	41.7 (0.91, >999)	0.056
Interfollicular CD10	Positive vs Negative	11.06 (1.74‐70.41)	0.011
FLIPI	Intermediate vs Low	2.21 (0.20‐24.17)	0.096
	High vs Low	10.35 (0.75‐142.85)	

## 4 DISCUSSION

In the present study, POD within 2 years after study entry in patients receiving frontline rituximab‐based biologic noncytotoxic therapy is associated with an inferior survival. To our knowledge, this is the first study of firstline immunotherapy regimens to evaluate the association of early progression with survival.

The 2‐year progression‐ and event‐free survival (PFS24 or EFS24) from diagnosis is becoming established as an important dynamic prognostic tool for patients.^4^, ^5^, ^6^ Similar to the LymphoCare,^4^ the German Low‐Grade Lymphoma Study Group (GLSG), and British Columbia Cancer Agency (BCCA) analysis of frontline follicular lymphoma patients,^5^ 17% of our patients had POD within 24 months from diagnosis and 5‐year OS was 69% in this group.

We also evaluated POD from study entry in these trials. Date of study entry was a more robust and accurate time‐point in patients enrolled on these 3 CALGB studies, whereas date of diagnosis may have been less accurate in patients who were initially observed without therapy for several months or years until study enrollment. When evaluating the PFS24 from study entry in our analysis, 28% of patients met the early POD criteria, and 5‐year OS was 74% in this group, similar to the OS in patients with early POD defined as from the date of diagnosis to progression. Our results show that both PFS24 from date of diagnosis and from date of study entry are associated with overall survival.

In multivariable analysis, male sex, high FLIPI, low albumin, low absolute monocyte count, and interfollicular CD10 staining were all associated with early POD. The association of male sex with early POD and subsequent worse OS suggests a different biology of follicular lymphoma and different pharmacokinetics of rituximab in males compared to females.^15^ Hypoalbuminemia and low absolute monocyte count have been correlated with prognosis in other studies in FL.^16^, ^17^ We hypothesize that FL behaves more aggressively in patients with lower monocyte immunity, perhaps by suppression of monocytes by the lymphoma itself. Lastly, interfollicular CD10 by immunohistochemistry was identified by Fouad‐Younes et al^18^ as correlating with OS in FL, and this study confirms these findings, specifically associating it with early POD as well. The presence of CD10^+^ B‐cells in the interfollicular zones could indicate a more aggressive disease with extension beyond the follicles. The other immunochemistry markers studied, such as PD1 and FoxP3, did not separate a group more prone to early POD. In fact, it was previously reported that rituximab may circumvent the prognostic significance of these IHC markers.^19^ More investigation into the molecular and genetic biology of early POD FL is still needed and may ultimately help to identify these high‐risk patients at diagnosis.

Although eligibility criteria were mainly uniform, this study is limited by the heterogeneity of treatments of rituximab‐containing “doublets.” Both galiximab and epratuzumab are no longer being developed, and it is unclear what benefit was gained with these combinations compared to single‐agent rituximab. In single‐agent rituximab studies such as the RESORT and the SAKK35/98 studies, 25%‐33% have relapsed or progressed within 2‐3 years.^7^, ^8^ When compared to the 42% early progression rates with R‐galiximab and 26% in the R‐epratuzumab trial, the early relapse rates with single‐agent rituximab appear similar or even lower, suggesting that the anti‐CD80 and anti‐CD22 antibodies added little and may have even adversely impacted the efficacy of rituximab, although the study population on these two doublet trials likely had higher risk FLIPI scores at enrollment. Therefore, although these two antibodies are no longer utilized to treat FL, the 26%‐42% rates of early progression observed with these immunotherapy combinations are likely similar to the rates of early progression observed in patients treated with rituximab alone. As a result, this study suggests that inferior OS (5‐year OS 74%) is also expected in FL patients progressing within 24 months of firstline single‐agent rituximab, similar although perhaps not quite as poor as the inferior outcomes (5‐year OS of 50%) observed in early progressors after R‐CHOP, R‐CVP, and R‐FND.^4^, ^5^, ^6^

On this trial, the numbers of early progressors from the R‐galiximab (42%, CALGB 50402) and R‐epratuzumab (26%, CALGB 50701) studies were higher than observed with R‐lenalidomide (14%, CALGB 50803) and R‐CHOP (19%, National LymphoCare Study^4^), perhaps in part due to variability in the FLIPI scores of the patients enrolled on these trials, the variability in histology (FL grade 1‐2 vs 3), and the intensity of initial treatment. The number of high‐risk patients with FLIPI scores of 3‐5 was 37.3%, 30.5%, 3.5%, and 44% on CALGB 50402, CALGB 50701, CALGB 50803, and the National LymphoCare Study, respectively. Both the National LymphoCare Study and this study did demonstrate an association with high‐risk FLIPI score and early progression. Additionally, CALGB 50402, 50701, and 50803 all restricted enrollment of patients to grade 1‐2 or 3a FL at diagnosis. In the National LymphoCare study, transformed FL was excluded; however, FL grade 3 patients including 3a and 3b subtypes were enrolled. Thirty‐eight percent of patients on the National LymphoCare Study had grade 3 disease, compared to 5%‐10% of patients on the CALGB trials. As some patients with grade 3 disease may have prolonged remissions with anthracycline‐based treatment, it is possible treatment with R‐CHOP resulted in fewer early progressors than observed with the less intensive regimens in this subgroup. Lastly, the limited efficacy of the galiximab and epratuzumab compared to lenalidomide or CHOP also likely resulted in more early progressions on CALGB 50402 and 50701 than observed with R‐lenalidomide or RCHOP. Overall, although more patients experienced early progression with immunotherapy than observed with R‐chemotherapy, this trial demonstrates that even with less intensive immunotherapy, the application of PFS24 predicts overall survival and validates the use of this prognostic indicator across clinical trial settings for patients with FL.

Optimal treatment approaches for follicular lymphoma patients that progress within 2 years are not known. For early progressors after R‐chemotherapy, the role of high‐dose therapy (HDT) with autologous stem cell rescue for early progressors has not been well studied but is a commonly used strategy. Although HDT with autologous stem cell rescue can be an effective strategy for salvage,^20^ other less aggressive options may be as effective including new CD20 targeted agents such as obinutuzumab, and biologic agents, such idelalisib, copanlisib, or lenalidomide. The US National Clinical Trials Network recently activated the clinical trial S1608 to evaluate three different approaches for patients with a first relapse of FL within 24 months of completion of bendamustine‐based immunochemotherapy (with anti‐CD20 antibody therapy). In this Phase II trial, patients are randomized to obinutuzumab +CHOP, obinutuzumab +lenalidomide, or obinutuzumab +TGR1202 (a novel PI3K inhibitor). Hopefully, the results of this trial will clarify whether a more aggressive second‐line conventional immunochemotherapy approach or a novel targeted therapy approach is better for patients with early progression after immunochemotherapy.

In addition to better treatment options, identification of patients at risk for early POD is also needed. Assessment for minimal residual disease (MRD) using next‐generation sequencing (NGS) has shown early utility in follicular lymphoma.^21^ Biologic risk assessment at diagnosis using methods such as the m7 FLIPI may also help identify these patients at diagnosis and permit examination of novel frontline therapeutic approaches in this high‐risk group.

In summary, early POD within 2 years of study entry in our patient cohort treated with upfront rituximab doublets defines a subgroup of patients who are at greater risk of death. This combined analysis, coupled with the National LymphoCare Study, the German Low‐Grade Study Group and British Columbia Cancer Agency, and the Maurer EFS24 cohorts validate PFS24 as an important prognostic marker in FL regardless of initial treatment.

**ClinicalTrials.gov:** NCT00117975 (CALGB 50402), NCT00553501 (CALGB 50701), and NCT01145495 (CALGB 50803).

## CONFLICT OF INTEREST

None declared.
